# Needle length control and the secretion substrate specificity switch are only loosely coupled in the type III secretion apparatus of *Shigella*

**DOI:** 10.1099/mic.0.059618-0

**Published:** 2012-07

**Authors:** Da-Kang Shen, Nao Moriya, Isabel Martinez-Argudo, Ariel J. Blocker

**Affiliations:** 1 Schools of Cellular and Molecular Medicine and Biochemistry, University of Bristol, Bristol BS8 1TD, UK; 2 Graduate School of Frontier Biosciences, Osaka University, 1-3 Yamadoaka, Suita, Osaka 565-0871, Japan

## Abstract

The type III secretion apparatus (T3SA), which is evolutionarily and structurally related to the bacterial flagellar hook basal body, is a key virulence factor used by many Gram-negative bacteria to inject effector proteins into host cells. A hollow extracellular needle forms the injection conduit of the T3SA. Its length is tightly controlled to match specific structures at the bacterial and host-cell surfaces but how this occurs remains incompletely understood. The needle is topped by a tip complex, which senses the host cell and inserts as a translocation pore in the host membrane when secretion is activated. The interaction of two conserved proteins, inner-membrane Spa40 and secreted Spa32, respectively, in *Shigella*, is proposed to regulate needle length and to flick a type III secretion substrate specificity switch from needle components/Spa32 to translocator/effector substrates. We found that, as in T3SAs from other species, substitution N257A within the conserved cytoplasmic NPTH region in Spa40 prevented its autocleavage and substrate specificity switching. Yet, the *spa40*_N257A_ mutant made only slightly longer needles with a few needle tip complexes, although it could not form translocation pores. On the other hand, Δ*spa32*, which makes extremely long needles and also formed only few tip complexes, could still form some translocation pores, indicating that it could switch substrate specificity to some extent. Therefore, loss of needle length control and defects in secretion specificity switching are not tightly coupled in either a Δ*spa32* mutant or a *spa40*_N257A_ mutant.

## Introduction

*Shigella flexneri* is the causative agent of shigellosis, which causes 1.1 million deaths each year, particularly among children under 5 years of age in developing countries (Kotloff *et al.*, 1999). The type III secretion (T3S) system, a protein transport device used by many Gram-negative bacteria to inject effector proteins into the cytoplasm of eukaryotic cells, plays an important role in controlling host cell signalling, invasion and death during infection (Schroeder & Hilbi, 2008). The T3S system of *Shigella* is composed of approximately 50 proteins, including a specialized Mxi–Spa T3S apparatus (T3SA), four chaperones, three transcriptional activators, three translocators and approximately 25 effectors (Parsot, 2009). The *Shigella* T3SA consists of a cytoplasmic portion called ‘the bulb’, a basal body spanning the inner and outer membranes and a hollow needle protruding outside the bacterium (Blocker *et al.*, 1999). The T3SA are evolutionarily and structurally related to the bacterial flagellar hook basal body (Minamino *et al.*, 2008), their most conserved features being the inner-membrane protein export machinery and a sophisticated mechanism for control of needle or hook length (Cornelis, 2006).

The length of the flagellar hook is well regulated, although it differs somewhat from species to species (Hirano *et al.*, 1994; Shibata *et al.*, 2007). The length of the needle is tightly controlled to match specific structures at the bacterial and host-cell surfaces, ensuring efficient delivery of effectors into the host cell (Mota *et al.*, 2005; West *et al.*, 2005). It also varies between different bacterial species, for instance, 45 nm for *S. flexneri* (Tamano *et al.*, 2002) and 58 nm for *Yersinia enterocolitica* E40 (Journet *et al.*, 2003). Different mechanisms have been proposed to explain length control of flagellar hooks and virulence T3S needles. Two protein families, namely the FliK/YscP family and FlhB/YscU family, are always involved in these models. Together they regulate a substrate specificity switch, which leads to the arrest of hook/needle growth and hence determines hook/needle length (Botteaux *et al.*, 2008; Cornelis, 2006; Erhardt *et al.*, 2011; Ferris & Minamino, 2006; Ferris *et al.*, 2005; Fraser *et al.*, 2003; Journet *et al.*, 2003; Makishima *et al.*, 2001; Marlovits *et al.*, 2006; Minamino & Macnab, 2000; Moriya *et al.*, 2006; Wagner *et al.*, 2010). What remains unclear is how these two components function at a mechanistic level.

The FliK/YscP family members are elongated, soluble proteins showing some structural disorder (Minamino *et al.*, 2004; Mizuno *et al.*, 2011) and carrying a more stably folded type III secretion substrate specificity switch domain (Agrain *et al.*, 2005; Minamino *et al.*, 2006). These proteins, including Spa32 in *Shigella*, may function as a molecular ruler or ‘tape measure’ that physically samples needle lengths as the proteins are secreted through the needle channel in low numbers and also to the more abundant needle subunits during needle growth (Botteaux *et al.*, 2008; Erhardt *et al.*, 2011; Journet *et al.*, 2003; Magdalena *et al.*, 2002; Moriya *et al.*, 2006).

**Fig. 1.**
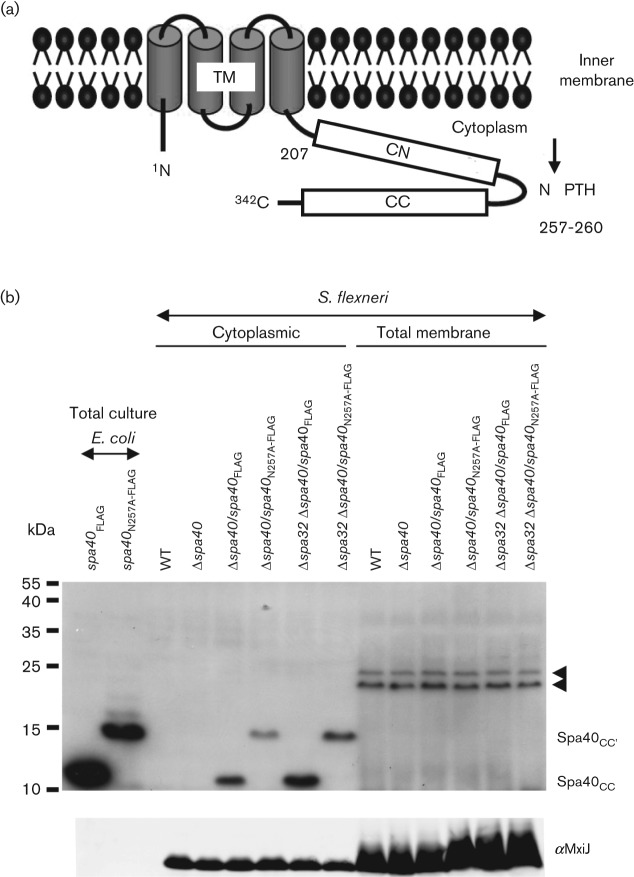
(a) Schematic representation of Spa40 based on previous studies (Deane *et al.*, 2008). Letters indicate N terminus (N), C terminus (C), conserved NPTH sequence, transmembrane domain (TM, residues 27–204), N-terminal half of the cytoplasmic domain (CN, residues 207–257) and C-terminal half of the cytoplasmic domain (CC, residues 258–342). Numbers indicate amino acid positions in Spa40 of *S. flexneri* 5a (NCBI: NP_085319). The black arrow represents the cleavage site in the NPTH region. (b) Analysis of the cleavage of Spa40 protein. Overnight total cultures of *E. coli* expressing Spa40_FLAG_ or Spa40_N257A-FLAG_ and the cytoplasmic and total cell membrane fractions of S. *flexneri* were analysed by immunoblotting with anti-FLAG antibodies (top) and anti-MxiJ (bottom). Two different forms of Spa40 are indicated on the right side (top) as follows: Spa40_CC_ and Spa40_CC′_. The latter results from cleavage at the alternative site. Non-Spa40-specific bands (black arrowheads) were also detected by the anti-FLAG antibody in Δ*spa40*. The data shown here are representative of results from two independent assays.

The FlhB/YscU protein family is one of the most highly conserved of all T3S protein families. In their N termini, these proteins carry four transmembrane regions, which position them in the inner bacterial membrane. The homologies amongst the C-terminal cytoplasmic domains of this protein family are particularly high (Allaoui *et al.*, 1994). Aligning the protein sequence of the FlhB/YscU family reveals the presence of a conserved 4 amino acid sequence, NPTH, in the middle of their C-terminal cytoplasmic domains (Allaoui *et al.*, 1994). The C-terminal cytoplasmic domain of the FlhB/YscU family undergoes autocleavage between the asparagine and proline residues within the NPTH sequence, leading to a small conformational change in the C-terminal domain, which may then interact with FliK/YscP family proteins via their type III secretion substrate specificity switch (T3S4) domain and contribute to the substrate specificity switch (Björnfot *et al.*, 2009; Deane *et al.*, 2008; Ferris *et al.*, 2005; Lavander *et al.*, 2002; Lorenz & Büttner, 2011; Lountos *et al.*, 2009; Minamino & Macnab, 2000; Mizuno *et al.*, 2011; Morris *et al.*, 2010; Sorg *et al.*, 2007; Wiesand *et al.*, 2009; Zarivach *et al.*, 2008). The FlhB/YscU homologue in *S. flexneri* is Spa40, a 342-residue polypeptide (Allaoui *et al.*, 1994; Fig. 1a), which undergoes autoproteolytic cleavage before P258 resulting in two subdomains, N-terminal cytoplasmic Spa40_CN_ and C-terminal cytoplasmic Spa40_CC_ (Deane *et al.*, 2008).

T3S systems from animal pathogenic bacteria secrete at least three different sets of substrates, including (i) proteins involved in the assembly of the periplasmic and extracellular needle portions, MxiI (Blocker *et al.*, 2001) and MxiH in *Shigella*, respectively, (Marlovits *et al.*, 2004; Tamano *et al.*, 2000) and Spa32, (ii) translocators, IpaD, IpaB and IpaC in *Shigella*, involved in the formation of the distal needle tip complex and then the translocon, the bacterial pore inserted into host membranes and used to translocate the protein effectors of virulence (Blocker *et al.*, 1999; Ménard *et al.*, 1993; Veenendaal *et al.*, 2007), and (iii) effector proteins, including early effectors, such as IpgD** of *Shigella*, which are involved in entry into polarized epithelial cells in the early stage of infection, and late effectors, which enable the bacteria to survive intracellularly, promote intra- and intercellular movement and modulate the host inflammatory response (Parsot, 2009). The secretion of needle components precedes translocators/early effector protein export (Magdalena *et al.*, 2002). Our recent data suggest that upon T3S activation translocators and early effectors are secreted in the same overall group, but one class after the other (Kenjale *et al.*, 2005; Martinez-Argudo & Blocker, 2010). Therefore, the T3S system of *Shigella* switches its substrate specificity over time from needle subunits and Spa32 (early substrates) to translocators and early effectors (here grouped as intermediate substrates). Late effector proteins (late substrates) are only synthesized after release of the intermediate substrate during activation (Parsot *et al.*, 2005).

In this study, we investigated the function of Spa40 autocleavage and how it might affect or be affected by Spa32. We find that Spa32 is not required for the cleavage of Spa40 but that the presence of Spa40 (but not its normal cleavage) contributes to the expression/stability of Spa32. We also show that a *spa40*_N257A_ mutant is severely impaired in the export of intermediate substrates but still exports early substrates. Accordingly, the *spa40*_N257A_ mutant makes somewhat longer needles and assembles only a few tip complexes. It therefore fails to insert translocators into host membranes, as measured by contact haemolysis. However, we find that Δ*spa32*, despite polymerizing very long needles, is better able to release intermediate substrates (although it cannot switch to their secretion efficiently) and causes weak haemolysis. Therefore, loss of needle length control and defects in secretion specificity switching are not tightly coupled either in a *spa32* null mutant or in a *spa40*_N257A_ mutant.

## Methods

### 

#### Bacterial strains and culture conditions.

Bacterial strains used in this study are listed in Table 1. *S. flexneri* strains were maintained and selected on Congo red (CR) agar plates (Meitert *et al.*, 1991) and grown at 37 °C (except for the temperature shift experiments) in trypticase soy broth (Becton Dickinson) supplemented with antibiotics where appropriate (100 µg ampicillin ml^−1^, 50 µg kanamycin ml^−1^, 10 µg chloramphenicol ml^−1^ and 10 µg tetracycline ml^−1^).

**Table 1.** *S. flexneri* strains used in this study

Strain	Genotype [strain; plasmid(s)]	Reference
WT	Wild-type M90T, serotype 5a	Sansonetti *et al.* (1982)
WT (pRK2)	Wild-type M90T; pRK2*mxiH*	Kenjale *et al.* (2005)
Δ*spa40*	Wild-type M90T containing an in-frame deletion in *spa40* ORF, corresponding to residues 6–338	This study
Δ*spa40*/*spa40*	Δ*spa40*; pUC19*spa40*	This study
Δ*spa40*/*spa40*_N257A_	Δ*spa40*; pUC19*spa40*	This study
Δ*spa40*/*spa40*_FLAG_	Δ*spa40*; pUC19*spa40*_FLAG_	This study
Δ*spa40*/*spa40*_N257A-FLAG_	Δ*spa40*; pUC19*spa40*_N257A-FLAG_	This study
Δ*spa32*	MJ321	Magdalena *et al.* (2002)
Δ*spa32* Δ*spa40*	MJ321Δ*spa40*	This study
Δ*spa32* Δ*spa40*/*spa40*_FLAG_	MJ321Δ*spa40*; pUC19*spa40*_FLAG_	This study
Δ*spa32* Δ*spa40*/*spa40*_N257A-FLAG_	MJ321Δ*spa40*; pUC19*spa40*_N257A-FLAG_	This study
WT *mxiH*	Wild-type M90T; pACT3*mxiH*	Shen *et al.* (2010)
Δ*spa40*/*spa40*_FLAG_ *mxiH*	Δ*spa40*; pUC19*spa40*_FLAG_, pACT3*mxiH*	This study
Δ*spa40*/*spa40*_N257A-FLAG_ *mxiH*	Δ*spa40*; pUC19*spa40*_N257A-FLAG_, pACT3*mxiH*	This study
Δ*spa32 mxiH*	MJ321; pACT3*mxiH*	This study
Δ*spa32* Δ*spa40 mxiH*	MJ321Δ*spa40*; pACT3*mxiH*	This study
Δ*spa32* Δ*spa40*/*spa40*_FLAG_ *mxiH*	MJ321Δ*spa40*; pUC19*spa40*_FLAG_, pACT3*mxiH*	This study
Δ*spa32* Δ*spa40*/*spa40*_N257A-FLAG_ *mxiH*	MJ321Δ*spa40*; pUC19*spa40*_N257A-FLAG_, pACT3*mxiH*	This study

#### Molecular cloning

All primers used in this study are listed in Table S1 (available with the online version of this paper) and all constructs were verified by DNA sequencing.

##### Knockout of *spa40*.

In-frame deletion of amino acids 6–338 encoded by *spa40* was carried out by using the λ Red system (Datsenko & Wanner, 2000). A kanamycin resistance cassette was amplified from plasmid pKD4 using the primers *spa40*-KO-kanF and *spa40*-KO-kanR and electroporated into *S. flexneri* wild-type carrying the Red recombinase to replace *spa40*, giving rise to Δ*spa40*. A tetracycline resistance cassette, amplified from strain TH2788 (Frye *et al.*, 2006) using the primers *spa40*-KO-tetF and *spa40*-KO-tetR, was used to replace *spa40* in *S. flexneri* strain Δ*spa32* (Magdalena *et al.*, 2002), giving rise to Δ*spa32* Δ*spa40*.

##### Mutagenesis of *spa40*.

We generated a point mutation (N257A) in the NPTH sequence of Spa40 using a two-step PCR strategy. In the first step, 5′ and 3′ fragments of *spa40* were amplified from pWR100 (Buchrieser *et al.*, 2000) by using the primer pairs *spa40*-F/*spa40*_N257A_-R and *spa40*_N257A_-F/*spa40*-R, respectively. In the second step, using the primer pair *spa40*-F/*spa40*-R, the mixture of 5′ and 3′ fragments was used as the template to amplify *spa40*N_257A_, which was then cloned into pUC19 by ligation to the *Pst*I and *Eco*RI sites of the polylinker. The resultant plasmid was transformed into Δ*spa40* to obtain Δ*spa40*/*spa40*_N257A_ (Table 1). The primers *spa40*-F and *spa40*-FLAG-R were used to obtain Δ*spa40*/*spa*40_N257A-FLAG_.

#### Analysis of protein synthesis and secretion.

Total levels of protein expression, leakage and CR induction were determined as previously described (Martinez-Argudo & Blocker, 2010). For the leakage assay, TCA-precipitated supernatants from exponentially growing bacteria (OD_600_ approximately 1) were used.

#### Pulse–chase, time-course experiments.

Bacteria were grown overnight at either 37 or 30 °C and then diluted 1 : 50 and either they were grown at constant temperature or growth was shifted from 30 °C to 37 °C. At 45 min, 1.5 h, 3 h and 22 h, both cells and supernatants, corresponding to same quantity of bacteria based on the OD_600_, were collected by centrifugation. Supernatants were further precipitated by using TCA. Finally, whole cells and TCA-precipitated supernatants were resuspended into SDS-loading buffer.

#### Antibodies and Western blotting.

The antibodies include the mouse mAbs against IpaB H16 (Barzu *et al.*, 1993), IpaC K24 (Phalipon *et al.*, 1992), IpgD (Blocker *et al.*, 1999) and FLAG M2 (Sigma) and the rabbit polyclonal sera against IpaD (Ménard *et al.*, 1993), MxiC (Martinez-Argudo & Blocker, 2010), Spa32 (Magdalena *et al.*, 2002) and MxiJ (Zenk *et al.*, 2007). The anti-Spa40 polyclonal antibodies were raised against Spa40_C_ (residues 207–342; Deane *et al.*, 2008) in rabbits (Eurogentec) and were then purified by using immunoaffinity (Harlow & Lane, 1988) using a CNBr-activated Sepharose 4B column (GE Healthcare) where the beads were covalently coupled to Spa40_C(N257A)_. Goat anti-mouse DyLight 800 (Fisher Scientific) or goat anti-rabbit Alexa 680 (Invitrogen) conjugates were used as secondary antibodies. The membranes were then visualized by using an Odyssey infrared imaging system (LI-COR Biosciences).

#### Preparation of total cell membranes.

To prepare total cell membranes, 500 ml exponentially growing bacteria (OD_600_ approximately 1) were harvested by centrifugation (20 min, 3170 ***g***, 4 °C) and washed once with Tris buffer (20 mM Tris, 150 mM NaCl, pH 7.4). Bacteria were resuspended in 10 ml Tris buffer containing one tablet of protease inhibitor cocktail complete Mini, EDTA free (Roche) and 15 Kunitz units DNase I (Sigma) and disrupted twice by passage through a French press at 15 000 p.s.i. (103 500 kPa). After removal of unbroken cells by low-speed centrifugation (60 min, 6000 ***g***, 4 °C; twice), the supernatants were passed through a 0.45 µm filter. About 9 ml clarified lysates was deposited on the surface of 3 ml 10 % (w/w) sucrose in an ultracentrifuge tube (SW 41 Ti rotor) and centrifuged at high speed (60 min, 178 305 ***g***, 4 °C), the supernatants and the pellets were collected and an equivalent amount of protein from each was analysed by SDS-PAGE and Western blotting.

#### Electron microscopy.

To visualize needles at the cell surface of bacteria, ghost cells were obtained by osmotic shock treatment using glass beads as described previously (Kenjale *et al.*, 2005). Samples were deposited onto 300-mesh, freshly glow-discharged, Formvar and carbon-coated copper grids and subsequently stained for 1 min with 1 % (w/v) phosphotungstic acid at pH 7. Bacteria were visualized in a Tecnai12 transmission electron microscope (FEI) fitted with an FEI Eagle 4k×4k CCD camera at ×20 000 magnification using FEI Tecnai Imaging Analysis (TIA) software. The length of the needles was measured using a ruler on a large computer screen.

#### Contact haemolysis.

These assays were performed as described previously (Blocker *et al.*, 1999).

#### Red blood cell membrane isolation.

This assay was performed as described previously (Shen *et al.*, 2010).

#### Needle purification.

To overexpress the needle protein MxiH, the *mxiH* gene was amplified by PCR using *Shigella* virulence plasmid pWR100 (Buchrieser *et al.*, 2000) as template and primers *mxiH*-RBS and *mxiH*-*Hin*dIII. The PCR product was purified, digested with *Sac*I and *Hin*dIII and cloned into the IPTG-inducible plasmid pACT3 (Dykxhoorn *et al.*, 1996) giving rise to pACT3*mxiH*. Needles were purified as described previously (Veenendaal *et al.*, 2007), but using 100 µM IPTG (isopropyl-β-d-thiogalactopyranoside) to induce *mxiH* expression from pACT3*mxiH* (Shen *et al.*, 2010).

## Results

### Spa40_N257A_ cleaves differently to wild-type Spa40

To analyse the phenotype of a non-cleavable *spa40* mutant, we introduced a single point mutation in the NPTH sequence of Spa40 and expressed the resulting *spa40*_N257A_ *in trans* in *Escherichia coli* DH5α and *S. flexneri* Δ*spa40*. Using an affinity purified polyclonal anti-Spa40, we could easily detect Spa40 from *E. coli* B834 BL21(DE3) overexpressing Spa40_C_ (Deane *et al.*, 2008). However, we failed to detect full-length or cleaved Spa40 in either *E. coli* DH5α or *S. flexneri* expressing full-length Spa40 or Spa40_N257A,_ from low-/high-copy-number plasmids or the virulence plasmid. Yet, *in trans* overexpression of the full-length wild-type protein did not inhibit bacterial growth and did functionally complement a Δ*spa40* mutant (Fig. S1). This suggests that natively encoded Spa40 is expressed or stable only at very low levels in *Shigella* and that our anti-Spa40 is not sensitive enough to detect it. Therefore, we constructed C-terminally FLAG-epitope-tagged full-length *spa40*_FLAG_ and *spa40*_N257A-FLAG_ and expressed these *in trans* in *E. coli*, *S. flexneri* Δ*spa40* or Δ*spa32* Δ*spa40*.

Using an anti-FLAG antibody on total cultures of *E. coli* expressing Spa40_FLAG_, we detected a fragment of about 10 kDa, assignable to Spa40_CC_ after cleavage in the NPTH region (Fig. 1b, top). In contrast, in *E. coli* expressing Spa40_N257A-FLAG_, a protein fragment of about 15 kDa (indicated as Spa40_CC′_) was observed. However, no Spa40 products were detectable in total culture extracts of *S. flexneri* expressing Spa40_FLAG_ or Spa40_N257A-FLAG_ (not shown).

As cleaved YscU from *Y. enterocolitica* was enriched in bacterial membrane fractions (Sorg *et al.*, 2007), we prepared total cell membranes from *S. flexneri* and checked them using anti-FLAG antibodies. In both Δ*spa40* and Δ*spa32* Δ*spa40* expressing Spa40_FLAG_, a 10 kDa fragment corresponding to Spa40_CC_ was clearly detectable from the cytoplasmic but not the total membrane fraction. In addition, in both Δ*spa40* and Δ*spa32* Δ*spa40* expressing Spa40_N257A-FLAG_, a 15 kDa fragment corresponding to Spa40_CC′_ was exclusively detectable from the cytoplasmic fraction. However, we never detected full-length Spa40_FLAG_, which has a predicted size of 40.8 kDa. Lack of detection of full-length Spa40 in both *E. coli* and *S. flexneri* suggests that complete autocleavage occurred under these experimental conditions.

To verify that Spa40_CC_ was indeed enriched in the cytoplasmic fraction, we checked the fractionation of both MxiG and MxiJ, which are inner membrane components (Allaoui *et al.*, 1992, 1995; Blocker *et al.*, 2001) of the *Shigella* T3SA (Blocker *et al.*, 2001; Kubori *et al.*, 1998). Though we could always detect these proteins in the cytoplasmic fraction, there was a clear enrichment of MxiG (not shown) and MxiJ in the membrane fraction (Fig. 1b, bottom), supporting the notion that, when *spa40* is expressed *in trans* in *Shigella*, the majority of Spa40_CC_ genuinely partitions in the cytoplasmic fraction and any fraction that becomes membrane-associated is not detectable with our experimental set-up.

Together, these data indicate that, in *Shigella* (i) the conserved Asn within the NPTH region is essential for the cleavage of Spa40 at this site, (ii) the cleavage is complete, (iii) Spa32 is not necessary for cleavage of Spa40 and (iv) probably the majority of overexpressed and cleaved Spa40_CC_ is not associated with bacterial membranes.

### The *spa40*_N257A_ mutant exports normal levels of Spa32 but more needle subunits and lower basal amounts of translocators/early effectors

**Fig. 2.**
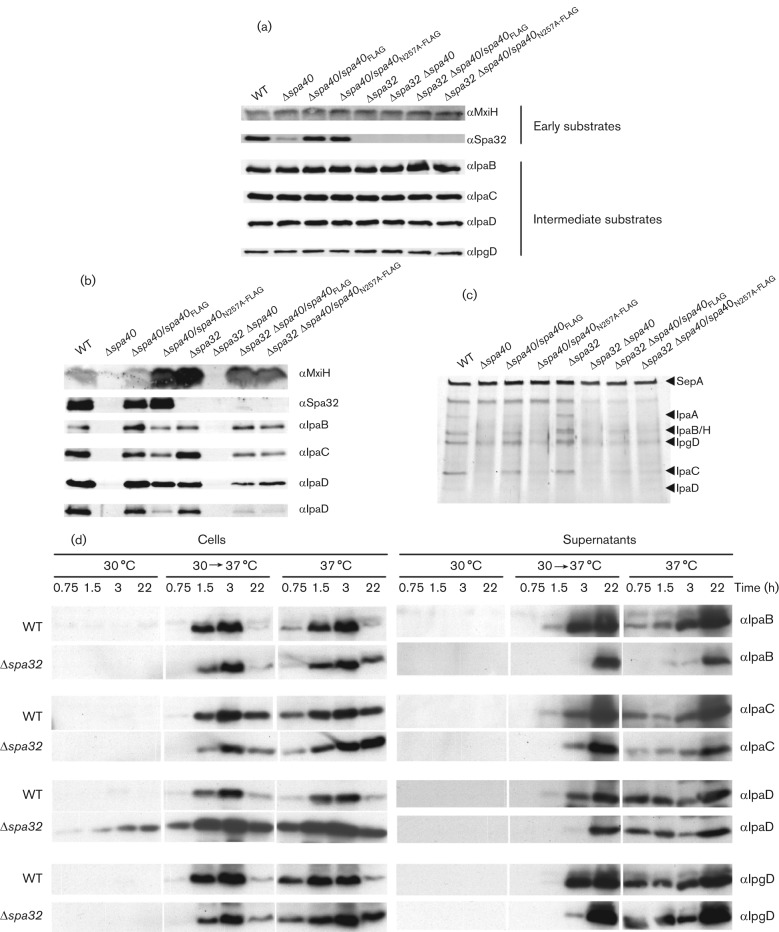
Expression and secretion profiles of *spa40* and *spa32* mutants. Total cultures (a) and TCA-precipitated supernatants of exponentially grown bacteria were analysed by immunoblotting (b), with antibodies indicated on the right. Supernatants were also checked by silver staining (c); the positions of the major Ipa proteins detected are indicated on the right. (d) Expression and secretion of Ipa proteins in Δ*spa32*, grown at 30 °C, 37 °C or shifted from 30 °C to 37 °C for 0.75, 1.5, 3 and 22 h, were analysed by immunoblotting. The wild-type (WT) was used as control and bacterial numbers were normalized by OD_600_. Each full set of WT and Δ*spa32* samples were run on separate gels but blotted in parallel. The antibodies used for the blots are indicated on the left. The data shown here are representative of results from two independent assays.

Next we tested whether secretion of translocators, early effectors and needle subunits were affected in the *spa40*_N257A-FLAG_ mutant. To make sure that the phenotypes observed were not due to alterations in the expression levels or stability of these proteins, we first analysed their levels in total culture by immunoblotting. All strains expressed similar levels of translocators (IpaB, IpaC and IpaD), early effector (IpgD) and needle subunit (MxiH) (Fig. 2a). However, the expression level of Spa32 was dramatically lower in Δ*spa40* compared with wild-type *Shigella* and Δ*spa40* complemented with either *spa40* or *spa40*_N257A_. This suggests that Spa40, but not its proper autocleavage, is required for intrabacterial expression/stability of Spa32.

To analyse the effect of the *spa40*_N257A_ mutation on the basal secretion of translocators, early effectors and needle subunits, the supernatants of bacteria grown to mid-exponential phase were analysed by silver staining and further verified by immunoblotting. As previously reported, the Δ*spa40* strain is defective in secretion, whereas complementation of Δ*spa40* with wild-type *spa40* restores secretion to levels similar to that of the wild-type (Fig. 2b, c; Botteaux *et al.*, 2010). In contrast, the *spa40*_N257A_ mutant secreted fewer translocators (IpaB, IpaC and IpaD) and early effectors (IpgD), but more needle subunits (MxiH; Fig. 2b, c), supporting the notion that Spa40 is involved in switching the T3S substrate specificity from early substrates, i.e., MxiH and Spa32, to intermediate substrates, i.e., translocators and early effectors. Fig. 2(b, c) shows that the level of secreted translocators and of the early effector IpgD are similar between wild-type and the Δ*spa32* strain, suggesting that, interestingly, Δ*spa32* has no defect in switching the secretion specificity from early substrates to intermediate substrates. However, Δ*spa32* does secrete much more MxiH, confirming that Spa32 is required for arrest of needle growth (Botteaux *et al.*, 2008; Magdalena *et al.*, 2002; Tamano *et al.*, 2002). Similar to Δ*spa40*, the Δ*spa32* Δ*spa40* strain is also defective in secretion. This indicates that, as expected, the known requirement of Spa40 for export apparatus function is dominant over lack of Spa32. No obvious secretion differences were observed between Δ*spa32* Δ*spa40* complemented either by *spa40* or by *spa40*_N257A_, and their basal secretion phenotype resembles that of Δ*spa40*/*spa40*_N257A_ more than that of Δ*spa32*. This further supports the notion that normal Spa40 cleavage is a prerequisite for the action of Spa32 in T3S maturation.

Together, these data suggest that in *Shigella*, Spa40, rather than Spa32, is the protein primarily mediating the secretion specificity switch from early to intermediate substrates and that its action requires correct autocleavage at the NPTH site.

### Δ*spa32* cannot switch to the secretion of translocators/early effectors efficiently

The Δ*spa32* and wild-type strains showed quite different phenotypes in terms of MxiH secretion (Fig. 2b) and needle length regulation (see later and Magdalena *et al.*, 2002). Thus, it is surprising that they secreted similar basal levels of translocators (except IpaD, which was reduced in Δ*spa32*) and of the early effector IpgD (Fig. 2b, c). We therefore asked whether the timing of Ipa ‘leakage’ is different between these two strains.

Since Ipas are expressed at 37 °C but not or less so at 30 °C (Le Gall *et al.*, 2005; Maurelli *et al.*, 1984), we tested for differences in secretion kinetics by shifting the cultures from 30 to 37 °C and performing a time-course analysis. Both cells and supernatants, corresponding to same quantity of bacteria, were collected and checked by Western blotting. At 30 °C, there was no expression of translocators (IpaBCD) or of the early effector IpgD in wild-type, as previously reported (Le Gall *et al.*, 2005; Maurelli *et al.*, 1984). However, we could easily detect the expression of IpaD from Δ*spa32* (Fig. 2d). Except for IpaD, the expression levels of translocators (IpaB and IpaC) and early effector (IpgD) were similar between wild-type and Δ*spa32*. However, the secretion of translocators, especially IpaD and IpaB and early effector IpgD, was clearly delayed in Δ*spa32*. These data suggest that Δ*spa32* has a delay, rather than an absolute defect, in switching the secretion from early substrates to intermediate substrates.

### The needle termination defect in Δ*spa32* is probably not due to retention of a needle assembly cap

In the flagellum, ‘assembly cap’ proteins are always attached at the distal end of the growing structure, including rod, hook and filament, to promote the efficient assembly of each substructure (Chevance & Hughes, 2008; Minamino *et al.*, 2008). Given the structural and mechanistic similarities between the flagellar hook and the T3S needle, it was expected that the needle would have an assembly cap too (Blocker *et al.*, 2003; Cornelis, 2006), which would be different to the ‘tip complex’ later assembled atop needles. If the growing needle did have a cap, such a cap should be found at the top of the continuously growing needles (Magdalena *et al.*, 2002) of Δ*spa32*. In addition, it might also be found in the Δ*ipaD* strain since IpaD is thought to be the first tip complex protein added (Veenendaal *et al.*, 2007), analogous to the first hook-junction protein in the flagellum. Lack of this latter protein also leads to assembly cap retention at the hook tip (Ohnishi *et al.*, 1994). Moreover, purified IpaD is able to halt needle growth *in vitro* (Poyraz *et al.*, 2010). However, we did not find evidence for the existence of an assembly cap protein using mass spectrometry analysis of needles from these strains (see Supplementary Methods and Results, and Table S2).

### *spa40*_N257A_ regulates needle length better than Δ*spa32* but both are unable to form substantial numbers of tip complexes

**Fig. 3.**
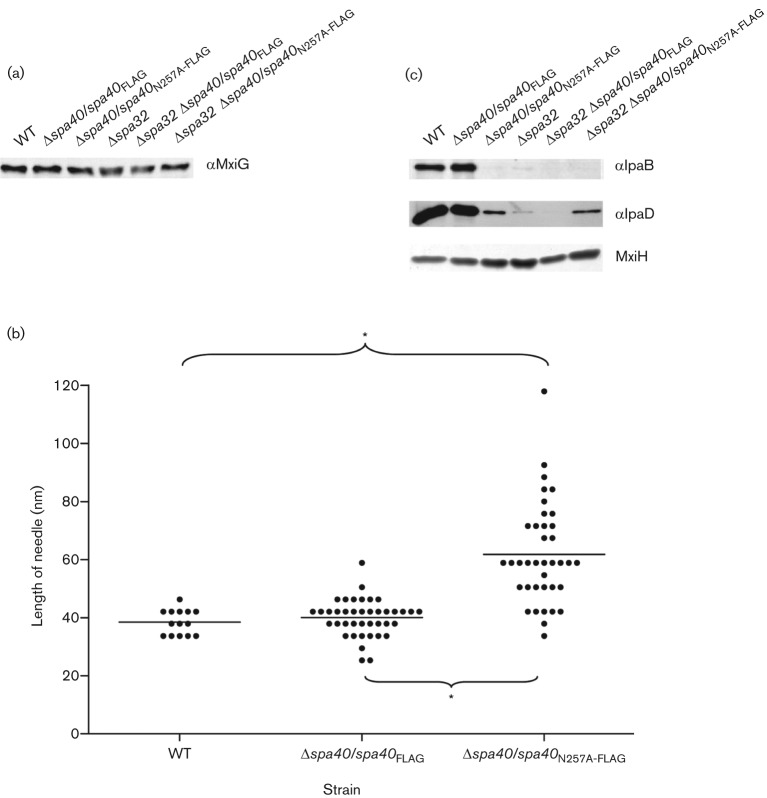
Analysis of needle-associated proteins and determination of needle length. (a) Overnight total cultures from strains overexpressing MxiH were analysed by immunoblotting with anti-MxiG antibodies. (b) Needle length determination. Data were analysed using a Kruskal–Wallis test and then by Dunn’s multiple comparison test. Dots represent individual needles. Data were based on two independent experiments. *P<0.001. (c) Needles from overnight-grown strains overexpressing MxiH were purified, normalized for the amount of MxiH by silver-stained SDS-PAGE (bottom) and analysed by immunoblotting with antibodies as indicated on the right. The data shown are representative of two independent assays giving similar results.

Loss of Spa32 causes hyperlong needles (Magdalena *et al.*, 2002). To assess the effect of Spa40_N257A_ on T3SA assembly, we first checked the expression level of MxiG, an inner membrane component of the needle complex. All strains showed similar levels of MxiG (Fig. 3a), suggesting that they have similar numbers of T3SA bases and hence needles. We next measured the length of needles from the wild-type and Δ*spa40* expressing Spa40 or Spa40_N257A_. Needles of the complemented Δ*spa40* strain had wild-type length, whereas needles from Δ*spa40* expressing Spa40_N257A_ were 50 % longer than that of the wild-type or the Δ*spa40* complemented strains (Fig. 3b) and had a broader length distribution. These data reflect the fact that the *spa40*_N257A_ mutant secreted as many or more needle subunits as Δ*spa32* (Fig. 2b). However, while Δ*spa32* and Δ*spa32* Δ*spa40* complemented with either *spa40* or *spa40*_N257A_ showed extraordinarily long needles up to 400–900 nm in length (Magdalena *et al.*, 2002; data not shown), the *spa40*_N257A_ mutant displayed only slightly extended needles. This indicates that Spa32 can still, albeit less efficiently, terminate needle length in the *spa40*_N257A_ mutant. This demonstrates that in *Shigella*, flipping of the early secretion specificity switch and needle termination are not tightly coupled events.

We previously reported that both IpaD and IpaB localize to the tip of mature, quiescent needles (Veenendaal *et al.*, 2007). Since the *spa40*_N257A_ mutant essentially cannot switch the T3S substrate specificity from needle to intermediate substrates and Δ*spa32* cannot do it efficiently (Magdalena *et al.*, 2002), we supposed that their needles might be immature and lack IpaD and/or IpaB. To facilitate examination of the Ipa composition of their needles, we overexpressed MxiH using the plasmid pACT3*mxiH* and prepared purified long needles as previously described (Shen *et al.*, 2010; Veenendaal *et al.*, 2007). The samples were normalized according to the amount of MxiH they contained and were then compared for Ipa composition by Western blotting (Fig. 3c). In needles derived from the complemented Δ*spa40* strain, IpaD and IpaB were found at levels similar to those found in wild-type needles. In contrast, very low IpaD and little IpaB were found in needles from *spa40*_N257A_ and Δ*spa32*, as well as Δ*spa32* Δ*spa40* complemented with either *spa40* or *spa40*_N257A_. Together, these data suggest that neither Δ*spa40*/*spa40*_N257A_ nor Δ*spa32* can assemble substantial numbers of mature needle tips including normal amounts of IpaD and IpaB.

### Both *spa40*_N257A_ and Δ*spa32* are non-inducible; however, Δ*spa32* is weakly haemolytic

**Fig. 4.**
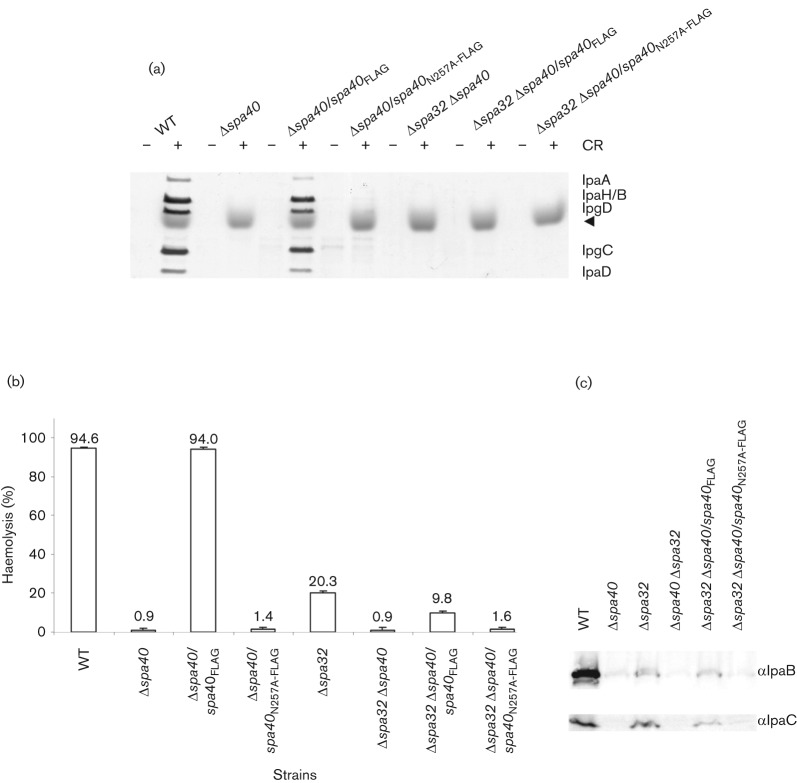
CR induction, haemolysis and membrane insertion of *spa40* mutants. (a) *spa40*_N257A_ mutant is uninducible by CR. Induced secretion of Ipa proteins after the addition (+) or in the absence (−) of CR was analysed by silver staining. The positions of the major Ipa proteins detected are indicated on the right. The bacterial numbers were normalized by OD_600_ and the data shown here are representative of two independent experiments giving similar results. A trace of CR is indicated by a solid arrowhead. (b) The haemolysis percentages of sheep RBCs are means±sd of results from two independent experiments performed in triplicate. (c) Insertion of IpaB and IpaC into sheep RBC membranes after contact haemolysis. The antibodies used for the Western blots are indicated on the right and the blots shown are representative of two independent assays giving similar results.

CR, a small amphipathic dye molecule, induces enhanced secretion of Ipa proteins in the wild-type *Shigella* (Bahrani *et al.*, 1997). In contrast, the *spa40*_N257A_ mutant was uninducible by CR (Fig. 4a). In addition, as previously reported, Δ*spa32* was also uninducible (Magdalena *et al.*, 2002; data not shown).

Haemolysis is the ability of *Shigella* to lyse red blood cells (RBCs) and is due to the formation of a 25 Å (2.5 nm) pore by IpaB and IpaC within the RBC membrane following contact induced with them during centrifugation (Blocker *et al.*, 1999). To study the effect of Spa40_N257A_ and the lack of Spa32 on pore formation, we investigated the contact haemolytic activity of their respective mutants. As expected, the Δ*spa40* strain was non-haemolytic, whereas complementation of Δ*spa40* with wild-type *spa40* restored haemolysis to levels similar to that of the wild-type (Fig. 4b). The *spa40*_N257A_ mutant was also non-haemolytic. Surprisingly, Δ*spa32* and Δ*spa32* Δ*spa40*/*spa40* caused 20.3 and 9.8 % haemolysis, respectively (Fig. 4b), despite their greatly reduced number of needle tip complexes (Fig. 3c). To determine whether Δ*spa32* and Δ*spa32* Δ*spa40*/*spa*40 could insert IpaB and IpaC within the RBC membrane, we examined the composition of the lysed RBC membranes isolated by floatation in a sucrose density gradient. As previously reported, IpaB and IpaC are present in these membranes when RBCs have been lysed by contact with wild-type bacteria (Fig. 4c; Blocker *et al.*, 1999). However, no IpaB and IpaC were detected from Δ*spa40*, Δ*spa32* Δ*spa40* and Δ*spa32* Δ*spa40*/*spa40*_N257A_, which all fail to cause haemolysis (Fig. 4b). In contrast, a little IpaB and IpaC were detected from Δ*spa32* and Δ*spa32* Δ*spa40*/*spa40*, which explains the observed 10–20 % haemolysis caused by these strains. Both Δ*spa32* and *spa40*_N257A_ can form small numbers of needle tips (see above). However, as only Δ*spa32* is able to switch the secretion specificity with low efficiency, only it can perform weak haemolysis. This weak inducibility is, however, not detectable (or not replicated) in the CR-induction assay.

## Discussion

The T3SA strongly resembles the hook basal body of the flagellar type III export apparatus in many respects and the two systems are assumed to share a mechanism for orchestrating T3S substrate specificity switching to control the length of the needles or hooks, respectively (Cornelis, 2006; Minamino *et al.*, 2008). The T3S substrate specificity switch depends on interactions between inner-membrane proteins belonging to the FlhB/YscU family and the T3S4 proteins belonging to the FliK/YscP family (Botteaux *et al.*, 2008; Ferris & Minamino, 2006; Minamino & Macnab, 2000). In this study, we investigated the role of the FlhB/YscU homologue Spa40 and the FliK/YscP homologue Spa32 of *S. flexneri* in the T3S substrate specificity switching and in the control of the needle length.

To understand the ordered export process, we introduced a point mutation into the NPTH cleavage site of Spa40. We found that the 40 kDa Spa40 is naturally cleaved and produces a 10 kDa CC fragment, as previously shown for FlhB (Minamino & Macnab, 2000) and for YscU (Lavander *et al.*, 2002; Sorg *et al.*, 2007). Though no 10 kDa CC fragment was detected from Spa40_N257A_, a band of 15 kDa was observed, suggesting that the *spa40*_N257A_ mutant cleaved itself at an alternative site. Alternative cleavage has also been observed in *Yersinia* spp. YscU mutants (Lavander *et al.*, 2002; Sorg *et al.*, 2007) and in the *Salmonella flhB*_N269A_ mutant, where it happens at D237/P238 (Fraser *et al.*, 2003). Though D237/P238 is highly conserved among the YscU/FlhB family (Zarivach *et al.*, 2008), the corresponding amino acids in *S. flexneri* are H225/F226. It seems unlikely that this could lead to the same cleavage mechanism (Zarivach *et al.*, 2008). If the alternative cleavage did occur at H225/F226 in Spa40_N257A_, the resulting C-terminal plus FLAG sequence would have a predicted molecular mass of 14.6 kDa, which corresponds to what we observed (Fig. 1b, top). As *flhB*_P238A_, which does not undergo cleavage at the secondary site, is wild-type for motility, cleavage at D237/P238 probably has no physiological significance (Fraser *et al.*, 2003).

In good agreement with the estimated stoichiometry of two FlhB molecules per flagellum (Zhu *et al.*, 2002), Spa40_CC_ could only be detected after enrichment. However, surprisingly, Spa40_CC_ was found in the cytoplasmic fraction rather in than membrane fraction. Although Cornelis’ group observed that cleaved YscU was enriched in total bacterial membranes under denaturing conditions, they did not mention whether they checked the cytoplasmic fraction (Sorg *et al.*, 2007). The enrichment of Spa40_CC_ in the cytoplasm might suggest that Spa40_CC_ is not tightly associated with the putatively membrane-bound N-terminal domain Spa40_CN_. Yet, the structures of the cytoplasmic domains of Spa40 (Deane *et al.*, 2008), *E. coli* EscU and *Salmonella typhimurium* SpaS (Zarivach *et al.*, 2008) highlight the tight association of the cleaved cytoplasmic subdomains. However, Deane *et al.* (2008) observed that the stable complex of Spa40_CN_–Spa40_CC_ was only formed under native conditions, indicating that the folded state of these proteins is essential for their tight association. Therefore, the cytoplasmic enrichment of Spa40_CC_ is most likely to be due to mistargetting and/or misfolding of the protein when expressed from a non-native promoter and/or at higher than native levels. Indeed, we did detect peptides corresponding to Spa40_CC_ in purified needle complexes (Zenk *et al.*, 2007; Cheung and other authors, unpublished data). As we know that FlhB_C_ is rapidly cleaved (Minamino & Macnab, 2000) and the cleavage of FlhB_C_ and its homologues is nearly complete (Fraser *et al.*, 2003; Lavander *et al.*, 2002; Lorenz & Büttner, 2011), this implies that, within the native T3SA base, the cleaved Spa40_CC_ is still attached to the inner membrane via its interaction with Spa40_CN_.

Our data show that cleavage at the NPTH sequence is required to mediate the secretion specificity switch from early (MxiH, Spa32) to intermediate substrates (translocators/early effectors), as reflected in the fact that *spa40*_N257A_ secreted more MxiH and made 50 % longer needles than the wild-type. This finding agrees with the previous report that *flhB*_N269A_ bacteria fail to switch from early rod-/hook-type substrate export to late filament-type substrate export (Fraser *et al.*, 2003). Though a *yscU*_N263A_ mutant prevents the export of translocators (Sorg *et al.*, 2007), it does not affect switching from needle subunits to Yop effectors, whereas *spa40*_N257A_, although it can still leak early effectors, is unable to induce their secretion. Therefore, from this point of view, either *spa40*_N257A_ is different from *yscU*_N263A_ or induction of effector secretion by CR in *Shigella* and by Ca^2+^ removal in *Yersinia* are not equivalent phenomena.

It was previously reported that Δ*yscP* *Yersinia* strain secreted dramatically lower levels of translocators/effectors relative to the wild-type strain (Edqvist *et al.*, 2003). However, our data showed that Δ*spa32* leaks levels of translocators/early effectors similar to those of the wild-type although it fails to switch the substrate specificity as quickly as the wild-type. In addition, although Δ*spa32* makes superlong needles, in *spa40*_N257A_ needle length is still approximately controlled by Spa32. The latter is reminiscent of the observation that the needle length in the *yscU*_N263A_ mutant can still be controlled by YscP (Sorg *et al.*, 2007), the only difference is that *spa40*_N257A_ secrete normal levels of Spa32 while *yscU*_N263A_ releases less YscP than wild-type bacteria do. This implies that (i) Δ*spa32*, unlike *spa40*_N257A_, can still switch secretion specificity from early to intermediate substrates and (ii) the FliK/YscP protein family might interact with somewhat different efficiency with the uncleaved FlhB/YscU protein family in different bacteria.

Although both *spa40*_N257A_ and Δ*spa32* present little IpaD and IpaB at their needle tips and are uninducible by CR, Δ*spa32* and Δ*spa32* Δ*spa40*/*spa40* cause 20.3 % and 9.8 % haemolysis, respectively. As haemolysis is due to the formation of a 25 Å (2.5 nm) pore by IpaB and IpaC within the RBC membrane, this suggests that Δ*spa32* does form some normal needle tip complexes but with very low efficiency. Weak haemolysis caused by Δ*spa32* also supports the observation that, unlike *spa40*_N257A_, Δ*spa32* has no absolute defect in switching the secretion specificity from MxiH to translocators. In fact, poor secretion of translocators, especially IpaC by *spa40*_N257A_ (Fig. 2b) and relatively good secretion of translocators, especially IpaC by Δ*spa32* (Fig. 2b, d), mirrors the haemolytic difference observed between *spa40*_N257A_ and Δ*spa32*. Why would Δ*spa32* make so few functional tip complexes (Fig. 3c) given it only displays a delay in secretion of the translocators, particularly IpaD (Fig. 2b–d)? IpaD is the first component of the tip complex, without which IpaB cannot bind to make it fully functional (Veenendaal *et al.*, 2007) in host-cell sensing. Therefore, a specifically greater reduction in IpaD release may enhance the kinetic block in tip complex assembly induced by the overall delay observed in translocator secretion in Δ*spa32*.

Loss of needle length control and failure to secrete translocators/effectors is dissociable in *Yersinia yscP* internal deletion mutants (Agrain *et al.*, 2005) but is tightly coupled in *Shigella spa32* internal deletion mutants (Botteaux *et al.*, 2008). Our observation is that loss of needle length control and defects in initial secretion of Ipas are not tightly coupled either in a *spa32* null mutant or in a *spa40*_N257A_ mutant. This agrees with the finding that *Salmonella fliK* deletion mutants are ‘leaky’ in that they produce approximately 90 % polyhooks and approximately 10 % polyhook-filaments (Patterson-Delafield *et al.*, 1973; Suzuki & Iino, 1981). Furthermore, our data indicate that the correct cleavage of Spa40 is vital for the substrate specificity switch, while Spa32 is mainly responsible for needle length control.

Accordingly, our data agree with the molecular tape measure model, which itself is based on the ruler model (Botteaux *et al.*, 2008; Journet *et al.*, 2003; Moriya *et al.*, 2006). The N-terminal end of the intermittently secreted FliK can bind to the hook cap strongly or to the wall of the growing hook, if for instance the hook is already too long to allow cap binding, with lower affinity (Minamino *et al.*, 2009). Its C terminus would remain inside the bacterial cytoplasm and interact with FlhB_C_ if the hook had reached a certain height, resulting in the switch of export specificity. As it has no assembly cap it can attach to, Spa32 would only bind to the wall of the growing needle and hence would still be able to regulate the length of the otherwise autonomously polymerizing needle. Lack of Spa32 would make needle length sensing and hence control impossible. However, in Δ*spa32*, Spa40 seems still functional for switching, albeit with poor efficiency. Therefore, Spa32 may only act to enhance a conformational change that occurs anyway autonomously in Spa40_C_.

Mizuno *et al.* (2011) solved the NMR structure of the FliK T3S4 domain. Based on functional data obtained from deletion mutants within FliK_C_, they constructed a model of the interaction between FliK_C_ and FlhB_C_ where the autocleaved NPTH sequence in FlhB contacts loop 2 of FliK_C_, perhaps triggering the switching event. In their model, this contact is sterically prevented when NPTH is not cleaved. However, measuring the interaction between FliK and FlhB by optical biosensing methods, Morris *et al.* (2010) found that, while the affinity between the two components is in the micromolar range, FliK binds to both wild-type (autocleaved) and mutant (non-cleaved) FlhBs with similar strength. These latter data support our findings that the activities of Spa32 and Spa40 are not tightly linked. However, key questions remain. When and how does Spa40 switch the substrate specificity, in the presence and absence of Spa32? How can Spa32 alone roughly determine needle length and then terminate needle growth in *spa40*_N257A_?
